# Myelodysplastic Syndrome and Autoimmunity: A Case Report of an Unusual Presentation of Myelodysplastic Syndrome

**DOI:** 10.1155/2011/560106

**Published:** 2011-09-22

**Authors:** Andrea L. Merrill, Hedy Smith

**Affiliations:** ^1^Tufts University School of Medicine, Boston, MA 02111, USA; ^2^Division of Hematology/Oncology, Tufts Medical Center, 800 Washington St, Boston, MA 02111, USA

## Abstract

Myelodysplastic syndrome (MDS) commonly presents asymptomatically or with symptomatic cytopenias. However, autoimmune phenomena in association with MDS have been well described in several case reports and case series. Typically, these autoimmune phenomena take the form of vasculitides, arthritis, connective tissue diseases, pulmonary infiltrates, or polymyalgia rheumatica. We present the case of a woman with MDS (karyotype 46,XX,+1,der(1;7)(q10;p10)[20], that evolved with an additional trisomy 8 clone) and a novel spectrum of autoimmune diseases including acute fibrinous and organizing pneumonia (AFOP) and lacrimal gland pseudotumor.

## 1. Introduction

The myelodysplastic syndromes (MDS) compose a heterogeneous group of clonal stem cell disorders, characterized by ineffective hematopoiesis in one or more cell lineages, and a propensity to progress to acute myeloid leukemia (AML). The risk of transformation to leukemia is determined in part by the degree of morphologic atypia, blast percentage in the bone marrow, and cytogenetics of the MDS clone [1, 2]. MDS patients are often asymptomatic, and the diagnosis is made at the time of routine laboratory screening tests that reveal cytopenias in one or more lines or dysplasia on the blood smear. Typical disease manifestations include fatigue and weakness from anemia, infections from neutropenia, or bleeding due to thrombocytopenia or platelet dysfunction. 

Autoimmunity in association with MDS is well described. Specifically, vasculitides, arthritis, connective tissue diseases, pulmonary infiltrates, polymyalgia rheumatica, and ulcerative colitis have preceded or accompanied the diagnosis of MDS in several case reports and series [3–6]. However, there are no cases, to our knowledge, of MDS presenting as acute fibrinous and organizing pneumonia (AFOP) and lacrimal gland pseudotumor. Here, we present a case of MDS manifesting as AFOP, lacrimal gland pseudotumor, polyarthritis, and fever of unknown origin.

## 2. Case Report

A 53-year-old postmenopausal woman, with a history of hypertension and fibromyalgia, presented with 1 month of intermittent chest pain, mild nonproductive cough, dyspnea on exertion, and a 22 lb weight loss over 4 months. Complete blood count revealed a macrocytic anemia with a mean corpuscular volume (MCV) as high as 108, with normal cobalamin and red blood cell folate. Coombs testing was negative, and her levels of lactate dehydrogenase (LDH), haptoglobin, and unconjugated bilirubin were normal. The peripheral blood smear showed dysplastic changes in all hematopoietic lineages including hypogranulation and hypersegmentation of the neutrophils and rare teardrop-shaped red cells, prompting a bone marrow evaluation. Bone marrow aspirate yielded a diagnosis of MDS, refractory anemia with excess blasts (RAEB-1; 8% blasts) with karyotype 46,XX,+1,der(1;7)(q10;p10)[20], resulting in the loss of 7q in 20/20 cells on examination of 20 metaphases. She required transfusion support for symptomatic anemia and became red blood cell transfusion dependent over the next several months.

As she presented with shortness of breath and pleuritic chest pain, a computed tomography (CT) scan of the chest was performed at initial presentation which revealed multiple, calcified, slightly enlarged mediastinal, and hilar lymph nodes as well as scattered ground-glass linear and nodular opacities. Bronchoalveolar lavage was suggestive, but not diagnostic, of alveolar proteinosis. Transbronchial biopsy and wedge resection by video-assisted thoracic surgery (VATS) procedure were also nondiagnostic. 

Two months later the patient presented with severe bilateral eye pain, marked eyelid swelling, high fever, and a blanching rash on the upper and lower extremities. Lacrimal gland biopsy showed prominent acute and chronic inflammatory infiltration with focal abscess formation, few macrophages, and perivascular inflammatory cuffing, diagnostic of lacrimal gland pseudotumor. Gram stain, acid-fast bacilli (AFB) stain, and fungal stains were negative. Skin biopsy revealed panicular hemorrhage without signs of panniculitis or vasculitis. Repeat chest CT showed increase in size and number of pulmonary nodules. Repeat VATS procedure with wedge resection of the lung now revealed a diagnosis of acute fibrinous and organizing pneumonia (AFOP) which is a rare form of acute lung injury distinct from both diffuse alveolar damage (DAD) and organizing pneumonia.

Initiation of intravenous Solumedrol 100 mg daily led to improved orbital and respiratory symptoms within 6 weeks. A repeat chest CT scan showed almost complete resolution of pulmonary nodules. Her transfusion requirements also declined while on high dose steroids. She was eventually placed on Prednisone 80 mg daily, which was decreased by 10 mg per week to a maintenance dose of 20 mg. Subsequently, treatment with Decitabine 20 mg/m^2^ (a hypomethylating agent) was initiated for her MDS. Four months later, while on Prednisone 20 mg daily and after completing her third cycle of Decitabine 20 mg/m^2^, she presented with high fevers, severe bitemporal headaches, and exertional dyspnea. Erythrocyte sedimentation rate (ESR) and C-reactive protein (CRP) were elevated to 140 mm/hr and 54.83 mg/L, respectively. A left temporal artery biopsy performed to assess for giant cell arteritis (GCA) was nondiagnostic. However, magnetic resonance imaging (MRI) of the head showed thickening of the temporal arteries. Blood and urine cultures were sterile. Antinuclear antibody (ANA), cytoplasmic antineutrophil cytoplasmic antibody (c-ANCA), and perinuclear cytoplasmic antibodies (p-ANCA) tests were negative. Chest CT scan performed due to increasing dyspnea showed recurrent pulmonary nodules. Intravenous Solumedrol 100 mg was resumed as there remained clinical concern for GCA. She experienced a brisk improvement in her symptoms, and her ESR decreased. She was discharged on Prednisone 100 mg with plan to taper the dose by 10 mg every 2 weeks.

Over the next few months, and as Prednisone was decreased to 40 mg daily, the patient presented with recurrent high fevers, flares of bilateral temporal headaches, and exquisite tenderness over the temporal regions. She also complained of blurred vision and jaw claudication during her second admission. Right temporal artery biopsy was again nondiagnostic for vasculitis. Extensive workup for infection, including CT scans, transesophageal echocardiogram, and tagged white blood cell scan were unrevealing. Repeat bone marrow aspirate and biopsy showed persistent MDS with the emergence of a new clone that expressed the trisomy 8 marker. Fluorescent in situ hybridization (FISH) detected nuc ish(D7S486x1)[151/200]/(D8Z2x3)[131/200]. There was doubling of the original abnormal clone in approximately 94 chromosomes. Aneuploidy of chromosomes 7q, 8, and 20q but not 5q was identified. Her symptoms again improved on high dose steroids. The patient was therefore started on oral methotrexate (MTx) 15 mg weekly, as she continued to manifest a dominant autoimmune phenotype. She defervesced after two doses, but MTx was promptly discontinued due to myelosuppression. 

In the next few months, she was repeatedly admitted to the hospital with fevers, pancytopenia, fatigue, severe myalgias, and arthralgias. A repeat bone marrow biopsy during her final hospitalization revealed markedly hypocellular bone marrow (<5%) without increase in white cell blasts. There was overall clinical deterioration due to bone marrow failure, progressive dyspnea and hypoxemia with progressive lung infiltrates, and intractable, generalized bone pain. The decision was made to institute comfort care measures, and the patient succumbed to her illness several days later. Postmortem exam was not allowed by the family.

## 3. Discussion

MDS is a common hematologic disorder that typically presents with one or more cytopenias. Autoimmunity is noted in up to 30% of patients in some series with autoimmune hemolytic anemia and immune thrombocytopenia seen most commonly [7]. Enright et al. studied 221 patients with MDS, of whom 30 (7.4%) had autoimmune organ disease [4]. In 7 cases, autoimmunity was diagnosed concurrently with MDS; 16 cases emerged along the MDS course, and 7 cases preceded the diagnosis. Autoimmune phenomena included cutaneous vasculitis, polyarthritis, myositis, polyneuropathy, glomerulonephritis, ulcerative colitis, pulmonary infiltrates, and pleural effusions [4]. Giannouli et al. also reported on 70 patients with MDS, of whom 12 (18.5%) developed rheumatologic syndromes including interstitial pulmonary infiltrates, polymyalgia rheumatica, and temporal arteritis [3]. A causal relationship is yet to be established between MDS and autoimmunity, but studies on the immunopathology and predictive biomarkers in MDS are ongoing. A number of immunoregulatory abnormalities have been described. Studies have shown that patients with MDS have reduced numbers of helper T cells [8], abnormal monocyte-derived dendritic cells [9], dysfunctional T regulatory cells [10], increased interferon regulatory factor-1 (IRF-1), m-RNA expression [11], decreased natural killer (NK) cell activity, and abnormalities of antibody-dependent cell killing [12].

Our patient presented with symptomatic anemia characteristic of MDS. The MDS clone was characterized by the 46,XX,+1,der(1;7)(q10;p10)[20] cytogenetic aberration that is reported in 1–3% MDS patients. By FISH, a trisomy 8 clone later emerged in a majority of 200 cells. Over the course of a few months, she developed histologically proven acute fibrinous and organizing pneumonia (AFOP), lacrimal gland pseudotumor and a rheumatologic syndrome of noninfectious fevers, polyarthritis, myositis, and presumed temporal arteritis. 

AFOP is a rare and relatively new histopathologic entity that is associated with clinical evidence of acute lung injury. The precise etiology is unknown but an association with collagen vascular diseases and malignancies has been reported [13]. A recent case report by Vasu et al. described a case of AFOP associated with Decitabine, which our patient also received for her MDS [14]. However, she was diagnosed with AFOP prior to receiving her first dose of Decitabine therapy. As far as we are aware, there are no reports of an association between MDS and AFOP, though there are case reports of pulmonary autoimmunity in patients with MDS. In the case series by both Giannouli et al. and Enright et al., noninfectious pulmonary infiltrates were reported as a manifestation of MDS [3, 4]. Additionally, Shimanuki et al. reported on a case report of organizing pneumonia in a patient with MDS [15]. Weng et al. also reported cryptogenic organizing pneumonia (COP) in a patient with MDS [16]. Response to steroids in all of these cases including our patient supports an immune pathogenesis. Interestingly, the der 1q; 7p chromosomal abnormality was previously described in 3 patients with MDS and disease-associated pulmonary syndromes [17].

Bilateral lacrimal gland pseudotumor is also rare in adults and is often recurrent and refractory to treatment. The etiology of orbital pseudotumor is unknown, but the proposed mechanisms include autoimmunity, infection, and abnormal wound healing [18, 19]. While there are no reports of MDS associated with lacrimal gland pseudotumor, there is a case report of MDS presenting with orbital inflammation. Rouhiainen described a patient who presented with chemosis, redness, and swelling of the eyelids that preceded an MDS diagnosis [20]. The orbital inflammation recurred twice over a 9-month period until finally controlled on a daily dose of maintenance prednisone.

 In summary, we report a case of MDS presenting with a novel spectrum of autoimmune diseases including AFOP and orbital pseudotumor. We speculate that these clinicopathologic entities extend the spectrum of autoimmune phenomenon seen in MDS and may be associated with an overall poorer prognosis. 
